# Long non-coding RNA LINC00174 promotes glycolysis and tumor progression by regulating miR-152-3p/SLC2A1 axis in glioma

**DOI:** 10.1186/s13046-019-1390-x

**Published:** 2019-09-06

**Authors:** Jian Shi, Yang Zhang, Bing Qin, Yongjie Wang, Xiangdong Zhu

**Affiliations:** Department of Neurosurgery, The Second Affiliated Hospital of Zhejiang University School of Medicine, No. 88 Jiefang Road, Hangzhou, 310000 China

**Keywords:** LINC00174, Glioma, SLC2A1, Glycolysis, miR-152-3p

## Abstract

**Background:**

Long non-coding RNA plays a crucial role in the occurrence and progression of glioma. We aimed to explore the function of LINC00174 in cell proliferation, apoptosis, migration, invasion and glycolysis of glioma cells, and investigate the molecular mechanism involved.

**Methods:**

LINC00174 expression in glioma tissues and peritumoral brain edema (PTBE) tissues was examined by RT-qPCR and in situ hybridization. The CCK-8, TUNEL, wound healing, transwell, and ELISA assays were performed to identify the effects of LINC00174 knockdown on cell viability, apoptosis, migration, invasion, and glycolysis, respectively. RNA immunoprecipitation, dual-luciferase reporter, RNA pull down, and western blot assays were performed to explore the molecular mechanisms of LINC00174 in glioma cells. A nude mouse xenograft model was used to investigate the role of LINC00174 in xenograft glioma growth.

**Results:**

LINC00174 was overexpressed in glioma tissues and cell lines. LINC00174 knockdown inhibited cell proliferation, migration, invasion and glycolysis of glioma cells, and LINC00174 exerted a tumorigenesis role. LINC00174 could interact with miR-152-3p/SLC2A1 axes. The miR-152-3p inhibitor or the SLC2A1 overexpression could rescue the anti-tumor effect of LINC00174 knockdown on glioma cells. Moreover, downregulation of LINC00174 also inhibited tumor volume and delayed the tumor growth in vivo.

**Conclusion:**

LINC00174 accelerated carcinogenesis of glioma via sponging miR-1523-3p and increasing the SLC2A1 expression, which could be considered as a molecular target for glioma diagnosis and therapy.

## Highlights

LINC00174 was overexpressed in glioma.

LINC00174 predicted an unfavorable prognosis in glioma patients.

LINC00174 promoted glycolysis and tumor progression by targeting miR-152-3p/SLC2A1 axes.

## Backgroud

Glioma is the most common primary brain tumor, which has the characteristics of high morbidity, high recurrence rate and high mortality, and seriously endangers people’s health [1]. It originates from astrocyte and oligodendrocyte, which are called astrocytoma and oligodendroglioma respectively [2]. The annual incidence of glioma in the population is 6.13/100,000 [3]. Although surgery combined with radiotherapy and chemotherapy can partly delay the recurrence time and prolong the survival time of glioma patients, the curative effect on some glioma patients with high malignancy is still unsatisfied. The median survival time of patients with WHO grade III degenerative astrocytoma and WHO grade IV pleomorphic glioblastoma was only 2 years and 1 year, respectively, even after surgery combined with radiotherapy and chemotherapy [3]. In the twentieth century, with the development of molecular biology and oncogenetics, people have a better understanding of the genetic characteristics of glioma, which provides a new idea for the subsequent gene therapy of glioma. Therefore, at present, the international community is focusing on the search for effective gene markers of glioma, hoping to be conducive to early intervention treatment and early prognosis judgment of glioma [4–6].

Long-chain non-coding RNA (lncRNA) is a kind of RNA with a length range of 200 nucleotides to 100,000 bases, which lacks an effective open reading framework and is transcribed by RNA polymerase II without protein coding function [7]. The main regulation modes of lncRNA include: (1) as a transcription regulator, interfering with the binding of transcription factors to promoters, interfering with gene transcription and chromatin remodeling; (2) as a regulator, influencing the transcription of target genes, up-regulating or down-regulating the expression of target genes; (3) binding to proteins through chaperone and regulating subcellular localization of proteins; and (4) binding to transcription to inhibit replication [8–10]. Through these pathways, lncRNA can play a variety of biological functions at the transcriptional level, post-transcriptional level and epigenetic level. Recent studies have shown that abnormal expression of lncRNA may affect the occurrence and development of glioma. A lot of lncRNAs have been reported to accelerate the tumorigenesis of glioma, including MALAT1 [11], SNHG16 [12], UCA1 [13]. According to the The Cancer Genome Atlas (TCGA) data analysis, we found the expression level of lncRNA LINC00174 was up-regulated in glioma tissue samples. Shen et al. reported that increased expression of LINC00174 was observed in colorectal cancer (CRC) tissues and cells, and LINC00174 indicated the poor prognosis of CRC patients [14]. However, the biological function of LINC00174 in the pathogenesis and development of glioma still remains unknown. Starbase predicted that LINC00174 could interact with miR-152-3p, which acts as an anti-cancer role in glioma [15, 16]. According to the prediction of Targetscan website, it was found that miR-152-3p can directly target Solution vector family 2 promotes glucose transporter 1 (SLC2A1), also known as glucose transporter 1 (GLUT1), which is a key protein in the energy metabolism pathway of cells [17]. SLC2A1 is overexpressed in several different types of cancer, including liver cancer, lung cancer, endometrial cancer, oral cancer, breast cancer, gastric cancer and glioma [18–21]. These observations suggest that SLC2A1 may be one of the driving genes in tumors. However, it is not clear whether LINC00174 can regulate the expression of microRNA-152-3p and SLC2A1 to play a role in glioma. Therefore, the objective of this study is to investigate whether LINC00174 promotes glycolysis and glioma progression by regulating the miR-152-3p/SLC2A1 axes.

In the present study, we explored the expression of LINC00174 in glioma tissues and normal tissues. The effect of LINC00174 on glioma progression was studied, and the underlying molecular mechanism by which LINC00174 regulated glioma cell phenotype was also investigated.

## Materials and methods

### Clinical tissue specimens

Forty-five paired brain glioma specimens and peritumoral brain edema (PTBE) tissues were collected from surgical tumor resections performed at The Second Affiliated Hospital of Zhejiang University School of Medicine (Hangzhou, China). Samples were collected between 2014 and 2017. Tissues were snap-frozen in liquid nitrogen and stored at − 80 °C for subsequent analysis. All tissues were obtained with written informed consent from each patient. The present study conformed to the ethical guidelines of the 1975 Declaration of Helsinki and was approved by the Institutional Ethical Review Committee of The Second Affiliated Hospital of Zhejiang University School of Medicine.

### Cell lines and cell culture

Human glioma cell lines (U251, LN229, H4, SW1783, and A172) and human embryonic kidney cell line HEK-293 T were purchased from Shanghai Institutes for Biological Sciences Cell Resource Center (Shanghai, China), while normal human astrocytes (NHAs) were purchased from Sciencell Research Laboratories (Carlsbad, CA, USA). HEK-293 T cells were cultured in Dulbecco’s modified eagle medium (DMEM, Life Technologies, Carlsbad, CA) supplemented with glucose and 10% fetal bovine serum (FBS; Life Technologies), glioma cells were cultured in Dulbecco’s modified eagle medium/F12 mixed medium supplemented with 10% FBS, and NHAs were cultured in astrocyte medium (Life Technologies). All cells were cultured at 37 °C in a humidified incubator containing 5% CO_2_.

### RNA isolation and reverse transcription-quantitative polymerase chain reaction (RT-qPCR)

Total cellular RNA was extracted using TRIzol reagent (Invitrogen, Thermo Fisher Scientifc, Inc.), according to the manufacturer’s protocol. For miRNA expression analysis, RT-qPCR was carried out by using the TaqMan MicroRNA Reverse Transcription kit, TaqMan Universal PCR Master Mix (Applied Biosystems; Thermo Fisher Scientific, Inc.). Relative miRNA expression levels were calculated as 2^-[(Ct of miRNA) – (Ct of U6)]^ after normalization to the expression of small nuclear RNA U6. Primers (RiboBio) used for stem-loop reverse-transcription PCR of miR-152-3p and U6 were as follows: miR-152-3p forward, 5′-AGGGTCAGTGCATGACAGA-3′ and reverse, 5′-TACCAACCAACCCACTCACT-3′; U6 forward, 5′-CGGGTGCTCGCTTCGCAGC-3′ and reverse, 5′-CCA GTGCAGGGTCCGAGGT-3′. For LINC00174 and SLC2A1 expression analysis, RT-qPCR was performed by using the TaqMan High-Capacity cDNA Reverse Transcription Kit, TaqMan Fast PCR Master Mix (Applied Biosystems; Thermo Fisher Scientific, Inc.) according to the manufacturer’s instructions with corresponding primers: LINC00174 forward, 5′-GGCCCAACACTTCCCTCAAA-3′ and reverse, 5′-CAGGGAGAAACGACCTGGAG-3′; SLC2A1 forward, 5′-AAGGTGATCGAGGAGTTCTACA-3′ and reverse, 5′-ATGCCCCCAACAGAAAAGATG-3′; β-actin forward, 5′-TCCTCTGACTTCAACAGCGACAC-3′ and reverse, 5′-CACCCTGTTGCTGTAGCCAAATTC-3′. Gene expression levels were normalized to β-actin expression and were calculated as 2^-[(Ct of GENES) – (Ct of β-actin)]^.

### Cell transfection

Two small interfering RNAs (siRNA) targeting LINC00174 (siLINC00174#1, and siLINC00174#2), and negative control RNAs (siNC) were generated in pLKO.1. Plasmid constructs were transfected into cells at 70–90% confluency using Lipofectamine 2000 (Invitrogen) and were then transfected again 24 h later. The siRNA targeting SLC2A1 (siSLC2A1), miR-152-3p mimics, inhibitors, and relative controls were obtained from GenePharma Co., Ltd. (Shanghai, China). Glioma cell transfection was conducted using Lipofectamine 2000 (Invitrogen) at a final concentration of 50 nM. To overexpress LINC00174, glioma cells were transfected with pcDNA3.1-LINC00174 using Lipofectamine 2000. After an additional 24 h, the transfected cells were collected and processed for further studies.

### Cell Counting Kit-8 (CCK-8) assay

Glioma cells (1 × 10^5^ cells per well) were seeded in 96-well plates and cultured for 24 h prior to analysis of cell proliferation using the CCK-8 (Dojindo Molecular Technologies, Gaithersburg, USA) assay. Cells were then cultured for a further 24, 48, or 72 h. Subsequently, all cells were incubated with 10 μL of CCK-8 solution at 37 °C for 4 h. To obtain cell growth curves, plates were read at 450 nm using a microplate spectrophotometer (Thermo Fisher Scientifc, Inc.). All experiments were performed in triplicate.

### TUNEL

An in-situ cell death detection kit (Roche, Basel, Switzerland) was used to measure cell apoptosis. Briefly, cells were blocked with H_2_O_2_ (3% in methanol) for 5 min and then labeled with TdT labeling reaction mix for 1 h at 37 °C. Nuclei exhibiting DNA fragmentation were visualized with 3,3′-diaminobenzidine (DAB) for 15 min and observed under a light microscope (Olympus Corporation,, Tokyo, Japan).

### Cell migration and invasion assay

Cell migration was evaluated using a wound-healing assay. In brief, 48 h after transfection, glioma cells were cultured in 6-well plates (5 × 10^4^ cells per well). After reaching 90–95% confluence, the monolayer of cells was scratched with a sterile plastic micropipette tip and cells were then cultured under standard conditions for 24 h. Following several washes, recovery of the wound was observed and imaged using an X71 inverted microscope (Olympus Corporation).

A transwell invasion assay was performed to assess cell invasion. Transfected cells (1 × 10^5^) were seeded into the upper chamber of Matrigel-coated inserts containing serum-free medium. Medium supplemented with 10% FBS (Life Technologies) was added to the lower chamber as a chemoattractant. Cells were then allowed to invade for 48 h at 37 °C with 5% CO_2_. Cells that invaded the lower chamber of the filter were fixed in 70% ethanol for 30 min and stained with 0.1% crystal violet for 10 min at 25 °C. The number of cells that migrated to the lower chamber was counted in five randomly selected fields under an X71 inverted microscope.

### Glucose uptake and lactate production assay

Glioma cells were cultured in glucose-free DMEM for 16 h, and then incubated with high-glucose DMEM under normoxic conditions for an additional 24 h. Culture medium was then removed, and intracellular glucose levels were measured using a fluorescence-based glucose assay kit (BioVision, Milpitas, California, USA) according to the manufacturer’s instructions. Lactate levels were measured using a lactate oxidase-based colorimetric assay read at 540 nm according to the manufacturer’s instructions (Beyotime, Wuxi, China) and normalized to cell number.

### Bioinformatics analysis

The target miRNAs of LINC00174 were predicted via computational algorithms, including starbase (http://starbase.sysu.edu.cn) and miRanda (http://www.microrna.org). The highest-ranked predicted target of LINC00174 was miR-152-3p. To identify genes targeted by miR-152-3p, we used the online programs, TargetScan (http://www.targetscan.org/) and miRanda (http://www.microrna.org). From the list of target genes obtained, all genes likely to contribute to glioma progression were extracted. The 3′-UTR of SLC2A1 was predicted to have miR-152-3p-binding sites.

### Luciferase reporter assay

To identify the LINC00174- and SLC2A1-binding sites in the miR-152-3p promoter, miR-152-3p promoter reporter constructs containing either wild-type, mutated LINC00174-binding sites, or mutated SLC2A1-binding sites were transfected with pRL-SV40 Renilla luciferase vectors into HEK293T cells using the LT1 Transfection Reagent (Mirus, Madison, WI, USA). Luciferase assays were performed using the Dual Luciferase Reporter Assay System (Promega, Madison, WI, USA) 48 h after transfection. Transfections were performed in triplicate, and measurements from transfections were analyzed after normalization to firefly luciferase activity.

### RNA pull-down assay

Biotinylated RNAs were transcribed using Biotin RNA Labeling Mix (Roche) and T7 polymerase (Promega) and subsequently treated with RNase-free DNase I (Promega) and RNeasy Mini Kit (Qiagen). Next, magnetic beads were added to each binding reaction sample and incubated at room temperature. Finally, the beads were washed, and eluted proteins were detected by RT-qPCR analysis.

### RIP analysis

RIP analysis was conducted in glioma cells using Magna RIP RNA-binding protein immunoprecipitation kit (Millipore, MA) according to manufacturer’s instructions. Briefly, cells were collected after washing with cold PBS and RIP lysis buffer was added. The suspension was then centrifuged and 100 μL from each cell lysate was transferred to the RIP immunoprecipitation buffer, which contained Ago2-conjugated magnetic beads and IgG as a negative control (Millipore, MA, USA). The magnetic beads were washed with RIP wash buffer and then incubated with proteinase K at 55 °C for 30 min. Subsequently, RNA was extracted for RT-qPCR analysis.

### In vivo xenograft experiments

Male BALB/c nude mice (6 weeks old, *n* = 6) were purchased from Beijing HFK Bioscience Co. Ltd. (Beijing, China) and were maintained under pathogen-free conditions. Animal experiments were approved by the Animal Care and Use Committee of The Second Affiliated Hospital of Zhejiang University School of Medicine and were performed in accordance with the relevant guidelines and regulations of the committee. For analysis of tumor propagation, 1 × 10^7^ U251 tumor cells, transfected with a short hairpin RNA (shRNA) targeting either LINC00174 (shLINC00174) or a negative control shRNA (shNC), were subcutaneously injected into BALB/c nude mice. Tumors were weighed 3 weeks after injection. Tumor volume was calculated at the indicated time points using the following formula: volume = πab^2^/6 (a, tumor length; b, tumor width). Ki67 levels were detected by immunohistochemical staining of tumors.

### Western blot analysis

Total protein lysates were resolved by 10% SDS-PAGE and transferred to polyvinyl difluoride membranes (EMD Millipore, Billerica, MA, USA). Following blocking with 5% nonfat dry milk in Tris-buffered saline containing 0.1% Tween-20 (TBS-T) for 30 min at 37 °C, membranes were washed four times in TBS-T and incubated with primary antibodies overnight at 4 °C. The primary anti-bodies: anti-SLC2A1 (Abcam, Cambridge, UK, ab190163, dilution: 1:1000), E-cadherin (Abcam, ab15148, dilution: 1:1000), N-cadherin (Abcam, ab202030, dilution: 1:1000), Vimentin (Abcam, ab8978, dilution: 1:800), Cleaved caspase-3 (Abcam, ab2302, dilution: 1:800), Cleaved caspase-9 (Abcam, ab2324, dilution: 1:1000), Bcl-2 (Abcam, ab32124, dilution: 1:800), and Bax (Abcam, ab32503, dilution: 1:800) were used. Following extensive washing, membranes were incubated with a horseradish peroxidase-conjugated goat polyclonal anti-rabbit IgG secondary antibody (cat. no. 7074; Cell Signaling Technology, Danvers, MA, USA), at a dilution of 1:2000, for 1 h at room temperature. Immunoreactivity was detected by enhanced chemiluminescence (Pierce; Thermo Fisher Scientific, Inc., Waltham, MA, USA) and visualized using a ChemiDoc XRS imaging system and analysis software (Bio-Rad Laboratories, Inc., Hercules, CA, USA). β-actin (Abcam, ab179467, dilution: 1:800) served as a loading control.

### Statistical analysis

All data are presented as the mean ± standard deviation (SD) from three independent experiments. The statistical analyses were performed by using SPSS 18.0 software (IBM, New York, USA). Differences between groups were analyzed by using Student’s t-test (two groups) or one-way ANOVA (multiple groups). Overall survival (OS) was defined either as the time from surgery to death, or the time from surgery to the date of the last recorded follow-up visit. A Kaplan-Meier curve was plotted for survival analysis, and the difference between the two groups was compared using a log-rank test. Spearman’s correlation analysis was used to determine the correlations between the levels of miR-152-3p and LINC00174/SLC2A1 in glioma tissues. *P* < 0.05 was considered statistically significant.

## Results

### LINC00174 was over-expressed in glioma tissues and cell lines

**Fig. 1:**
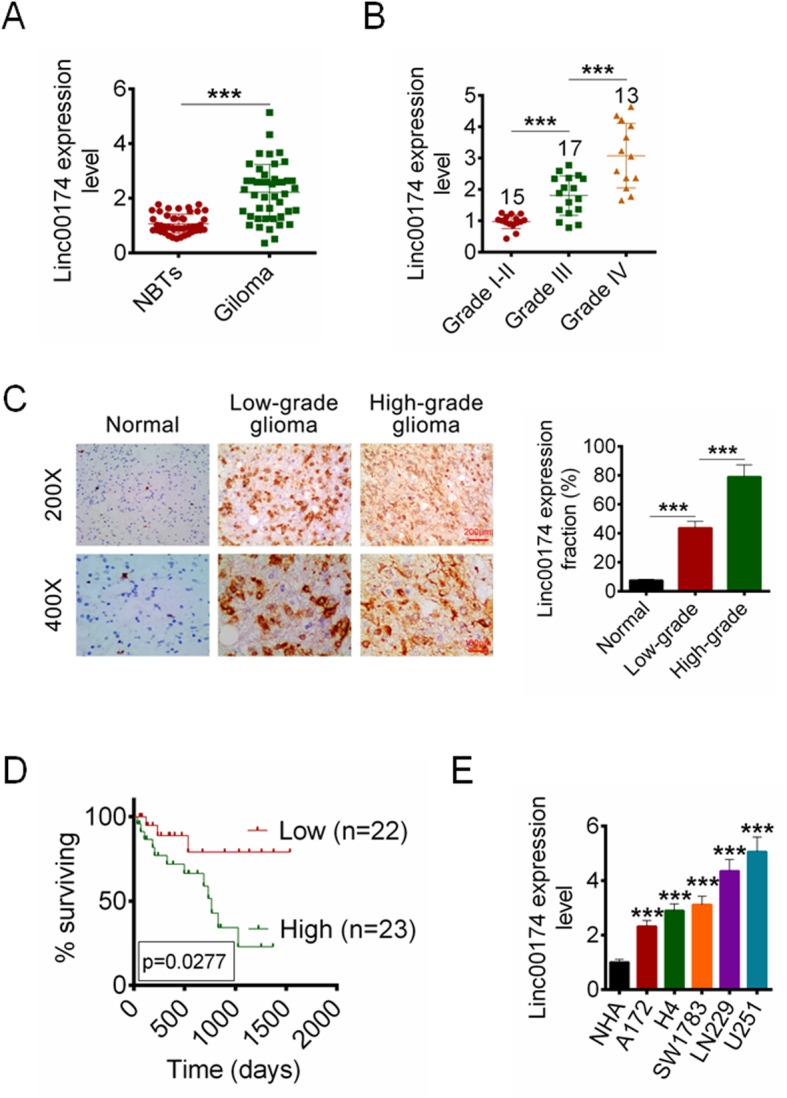
The expression of LINC00174 in glioma tissues and cell lines. **a** The expression of LINC00174 in PTBE and glioma tissues was identified by RT-qPCR. **b** LINC00174 expression in different grades of glioma patients was examined by RT-qPCR. **c** ISH was used for the LINC00174 expression detection in normal tissue, low-grade and high-grade of glioma tissues. **d** Survival rates of patients with glioma with high and low LINC00174 by Kaplan-Meier survival analysis. **e** The expression of LINC00174 in glioma cell lines and NHA cells was examined by RT-qPCR. Data are presented as the mean ± SD. ***P < 0.001 vs. NHA cells

We first identified the expression of LINC00174 in glioma tissues and normal tissues in 45 paired samples by RT-qPCR. The results showed that LINC00174 expression was increased significantly in glioma tissues compared with that in PTBE (Fig. 1a, *P* < 0.001). The expression of LINC00174 in different stages of glioma samples was examined by RT-qPCR and ISH analysis. As shown in Fig. 1b-c, the LINC00174 expression was higher in high-grade than that in low-grade. Furthermore, the high expression of LINC00174 predicted an unfavourable prognosis (Fig. 1d). The expression of LINC00174 in human astrocytes (NHA) and five glioma cell lines including U251, LN229, H4, SW1783, and A172 was also examined. The results showed that LINC00174 was overexpressed in glioma cell lines (Fig. 1e, *P* < 0.001).

### LINC00174 promoted cell proliferation of glioma cells in vivo and in vitro

**Fig. 2:**
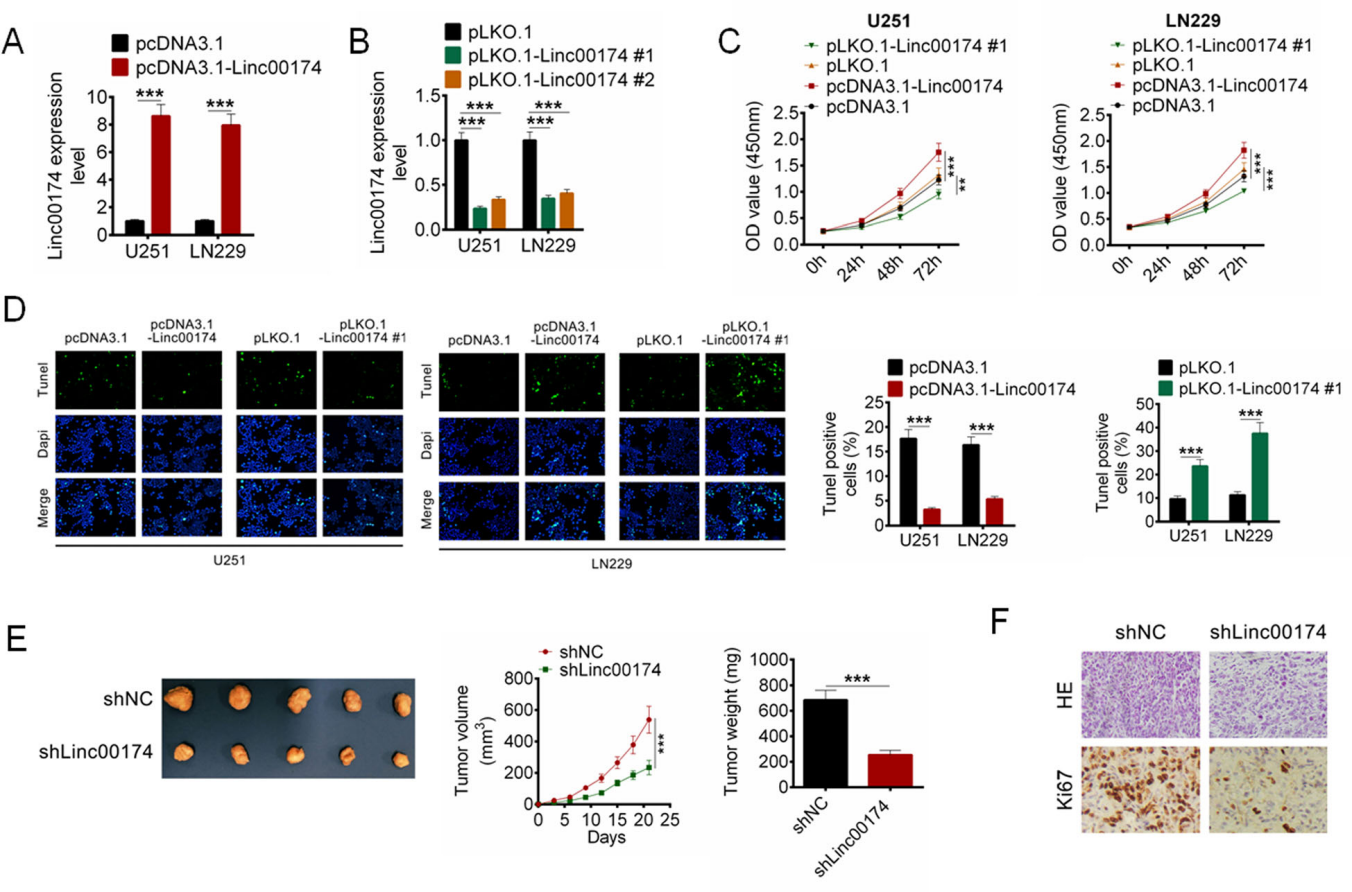
LINC00174 regulated cell proliferation and apoptosis in vitro and in vivo. **a** U251 and LN229 cells were transfected with pcDNA3.1 or pcDNA3.1-LINC00174, and LINC00174 expression was examined by RT-qPCR. **b** U251 and LN229 cells were transfected with pLKO.1, or pLKO.1-LINC00174#1, or pLKO.1-LINC00174#2, and LINC00174 expression was examined by RT-qPCR. **c** Cell proliferation was examined by CCK8 assay. **d** Cell apoptosis was identified by TUNEL analysis. **e** The effect of LINC00174 on tumor growth was examined by a nude-mouse transplanted tumor model. Tumor growth curves were established by measuring tumor volume every 3 for 21 days after injection. Tumor weights isolated from nude mice in each treatment group were determined on day 21 after injection. **f** Ki67 expression in tumor tissues were asses by IHC analysis. Data are presented as the mean ± SD. **P < 0.01, and ***P < 0.001

To identify the effect of LINC00174 on cell proliferation of glioma cells, U251 and LN229 cells were transfected with pcDNA3.1-LINC00174 or pLKO.1-LINC00174, or their relative controls. The transfection efficiency was then identified by RT-qPCR, and pLKO.1-LINC00174#1 with better efficiency for LINC00174 knockdown was used for the further studies (Fig. 2a-b, *P* < 0.001). Cell proliferation and apoptosis were then identified by CCK8 and Tunel, respectively. As shown in Fig. 2c-d, cell proliferation of U251 and LN229 cells with pcDNA3.1-LINC00174 transfection was promoted compared with that of pcDNA3.1 transfected cells (*P* < 0.001), and pcDNA3.1-LINC00174 also decreased cell apoptosis of glioma cells (*P* < 0.001). On the contrary, pLKO.1-LINC00174 transefection significantly inhibited cell proliferation and facilitated cell apoptosis of U251 and LN229 cells compared with that of pLKO.1 transfected cells (Fig. 2c-d, *P* < 0.001). Moreover, the effect of LINC00174 knockdown on tumor growth was also examined by a nude-mouse transplanted tumor model. The results exhibited that shLINC00174 obviously delayed tumor growth, decreased tumor volume, and reduced tumor weight compared with the shNC group (Fig. 2e, *P* < 0.001). The LINC0074 knockdown also effectively inhibited the expression of Ki67 in tumor tissues in comparison with that in tumor tissues of shNC group (Fig. 2f, *P* < 0.001).

### LINC00174 accelerated cell migration, invasion and glycolysis of glioma cells

**Fig. 3:**
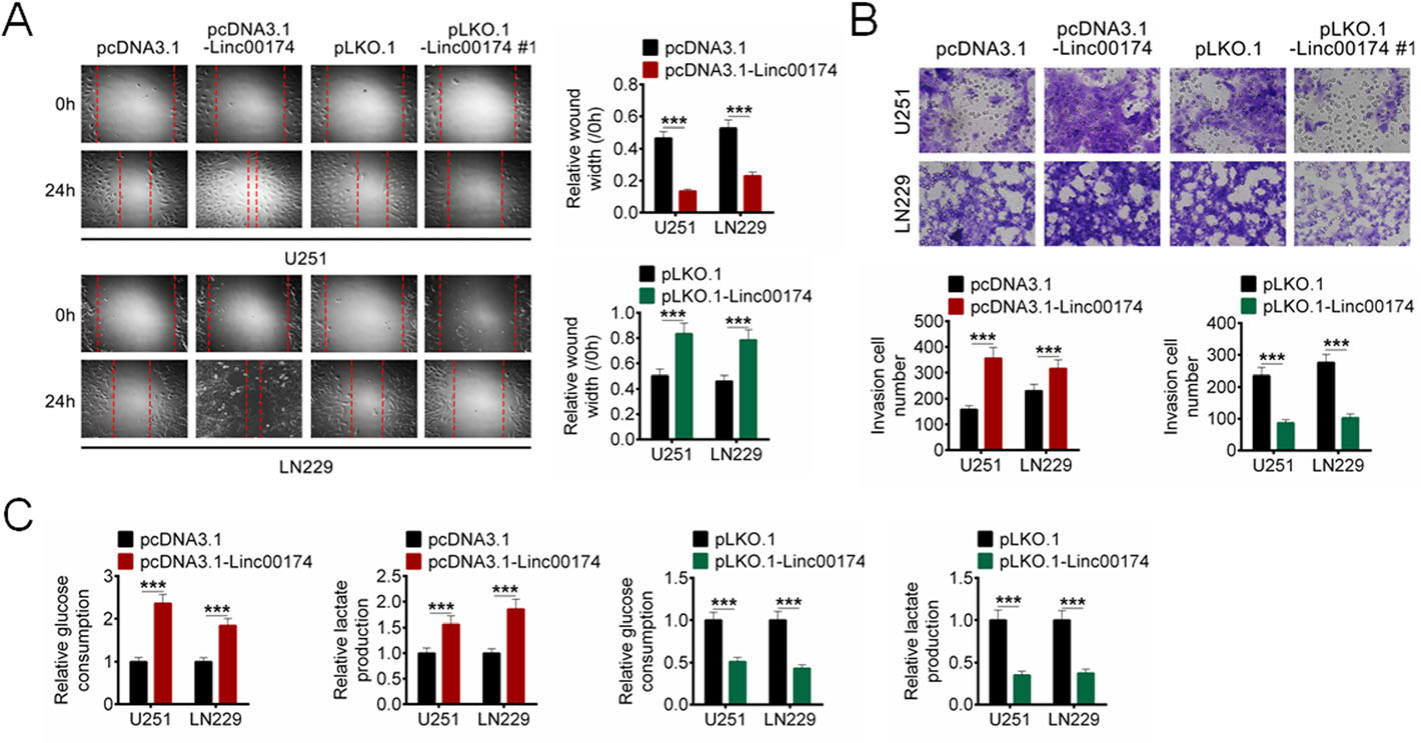
LINC00174 regulated cell migration, invasion and glycolysis of glioma cells. **a** The effect of LINC00174 on cell migration of glioma cells was evaluated by wound healing assay. **b** Cell invasion of glioma cells was identified by transwell assay. **c** Glucose consumption and lactate production in U251 and LN229 cells with LINC00714 overexpression or knockdown were detected by ELISA analysis. Data are presented as the mean ± SD. ***P < 0.001

To explore the effect of LINC00174 on malignant tumor cell phenotype of glioma, cell migration, invasion and glycolysis of U251 and LN229 cells with LINC00174 overexpression or LINC00174 knockdown were evaluated. We found that LINC00174 overexpression promoted cell migration and invasion of U251 and LN229 cells, and LINC00174 knockdown inhibited cell migration and invasion of glioma cells (Fig. 3a-b, *P* < 0.001). The effect of LINC00174 on glucose consumption and lactate production in U251 and LN229 cells was also identified. As shown in Fig. 3c, LINC00174 overexpression promoted the glucose consumption and lactate production (*P* < 0.001), while LINC00174 knockdown showed the opposite effect (*P* < 0.001).

### LINC00174 directly targeted miR-152-3p

**Fig. 4:**
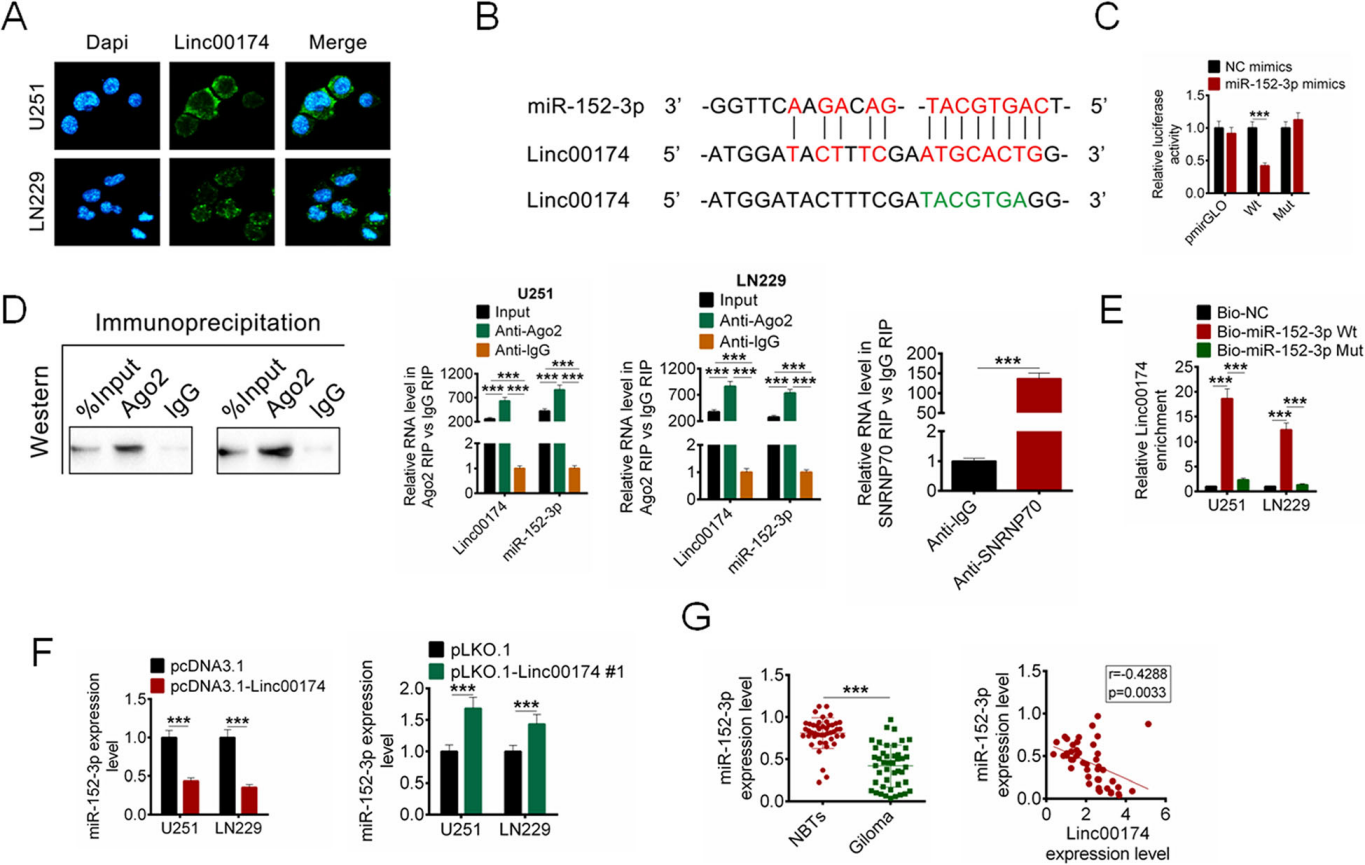
LINC00174 directly targeted miR-152-3p. **a** FISH analysis revealed that LINC00174 was located in cytoplasm. **b** The binding site of LINC00714 targeting miR-152-3p. **c**-**e** Dual luciferase reporter assay, RIP and RNA pull down were performed to identify LINC00174 directly combined with miR-152-3p. **f** The miR-152-3p expression in glioma cells with LINC00174 overexpression or knockdown was examined by RT-qPCR. **g** The expression of miR-152-3p in PTBE and glioma tissues was examined, and showed negative correlation with LINC00174 expression in glioma tissues. Data are presented as the mean ± SD. ***P < 0.001

To further explore the underlying mechanism by which LINC00174 facilitated cell proliferation, migration, invasion and glycolysis of U251 and LN229 cells, the targeted miRNAs of LINC00174 were predicted. By FISH analysis in Fig. 4a, the expression of LINC00174 mainly located in the cytoplasm. From the analysis of Starbase (http://starbase.sysu.edu.cn), miR-152-3p was predicted to combine with LINC00174, which was verified by luciferase reporter assay (Fig. 4b-c). Furthermore, immunoprecipitation was carried out to verify the bioinformatical prediction. Also, RIP assay was performed to unveil whether LINC00174 interact miR-152-3p directly. As shown in Fig. 4d, the enrichment levels of LINC00174 and miR-152-3p in Ant-Ago2 group were higher than that in Ant-IgG group, the results suggested that miR-152-3p could directly target LINC00174 (*P* < 0.001, Fig. 4d). By RNA pull down analysis, the LINC00174 enrichment in Bio-miR-152-3p-MUT showed no significance compared with the Bio-NC, while the LINC00174 enrichment in Bio-miR-152-3p-WT was significantly increased compared with the the Bio-NC (*P* < 0.001, Fig. 4e). The expression of miR-152-3p in U251 and LN229 cells with pcDNA3.1-LINC00174, or pLKO.1-LINC00174 or the relative controls transfection was examined by RT-qPCR. The results showed that miR-152-3p expression was decreased in U251 and LN229 cells transfected with pcDNA3.1-LINC00174, while increased in U251 and LN229 cells transfected with pLKO.1-LINC00174 (*P* < 0.001, Fig. 4f). Moreover, the expression of miR-152-3p in glioma tissues and PTBE was identified. The expression of miR-152-3p in glioma tissues was decreased significantly compared with that in the PTBE, and the expression of which exerted a negative correlation with LINC00174 expression in glioma samples (*P* < 0.001, Fig. 4g).

### miR-152-3p inhibited cell proliferation, migration and invasion through regulating SLC2A1 expression

**Fig. 5:**
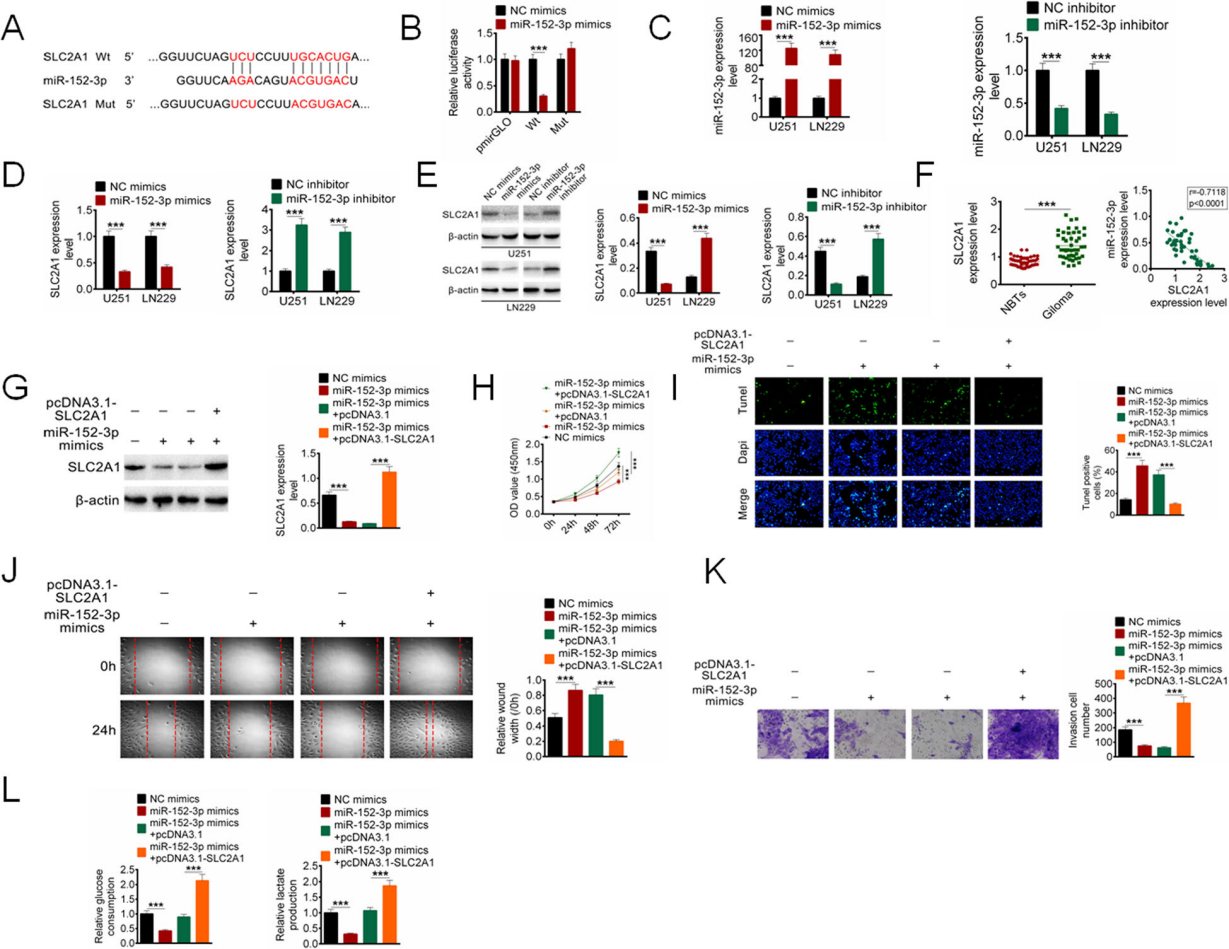
The miR-152-3p targeted SLC2A1 to regulated cell physiological activities of glioma cells. **a** miR-152-3p binding sites in SLC2A1 predicted by bioinformatics analysis. **b** Dual luciferase reporter assay was performed to identify SLC2A1 directly combined with miR-152-3p. **c** Glioma cells were transfected with miR-152-3p mimics or inhibitors. **d**-**e** The mRNA and protein expression of SLC2A1 expression in glioma cells with miR-152-3p mimics or inhibitors transfection was examined by RT-qPCR and western blot. **f** The SLC2A1 expression in glioma tissues was detected, and showed a negative correlation with miR-152-3p expression. **g** U251 cells were transfected with NC mimics, or miR-152-3p mimics, or miR-152-3p mimics + pcDNA3.1, or miR-152-3p mimics + pcDNA3.1-SLC2A1, SLC2A1 expression was examined by western blot analysis. **h**-**l** Cell proliferation, apoptosis, migration, invasion and glycolysis were then identified by CCK8, TUNEL, wound healing, transwell and ELISA, respectively. Data are presented as the mean ± SD. **P < 0.01, ***P < 0.001

We then explored the target mRNA of miR-152-3p through bioinformatics analysis. From the Targetscan (http://www.targetscan.org/vert_71/) analysis, SLC2A1 was predicted as the target of miR-152-3p (Fig. 5a). The interaction between miR-152-3p and SLC2A1 was then examined by luciferase reporter assay (Fig. 5b). U251 and LN229 cells were then transfected with miR-152-3p mimics or miR-152-3p inhibitors, and the transfection efficiency was evaluated by RT-qPCR (*P* < 0.001, Fig. 5c). The mRNA expression and protein level of SLC2A1 in U251 and LN229 cells with miR-152-3p mimics or miR-152-3p inhibitors transfection were identified by RT-qPCR and western blot, and the results showed that SLC2A1 expression was decreased in glioma cells with miR-152-3p mimics transfection, and increased in glioma cells with miR-152-3p inhibiors transfection (*P* < 0.001, Fig. 5d-e). Furthermore, SLC2A1 expression was increased in glioma tissues and negatively associated with miR-152-3p expression in glioma samples (*P* < 0.001, Fig. 5f).

The rescued experiments were performed, U251 cells were transfected with NC mimics, or miR-152-3p mimics, or miR-152-3p mimics + pcDNA3.1, or miR-152-3p mimics + pcDNA3.1-SLC2A1. SLC2A1 expression was increased in U251 cells with miR-152-3p mimics + pcDNA3.1-SLC2A1 transfection (*P* < 0.001, Fig. 5g). Cell proliferation, apoptosis, migration, invasion and glycolysis were then identified. As shown in Fig. 5h-l, cell proliferation, migration, invasion and glycolysis of U251 cells was significantly inhibited by miR-152-3p mimics compared with the NC mimics group (*P* < 0.001), cell apoptosis was increased in U251 cells transfected with miR-152-3p mimics (*P* < 0.001). While SLC2A1 overexpression rescued the anti-tumor effect of miR-152-3p on U251 cells (*P* < 0.001, Fig. 5h-l).

### LINC00174 exerted the oncogenesis role in glioma via targeting miR-152-3p

**Fig. 6:**
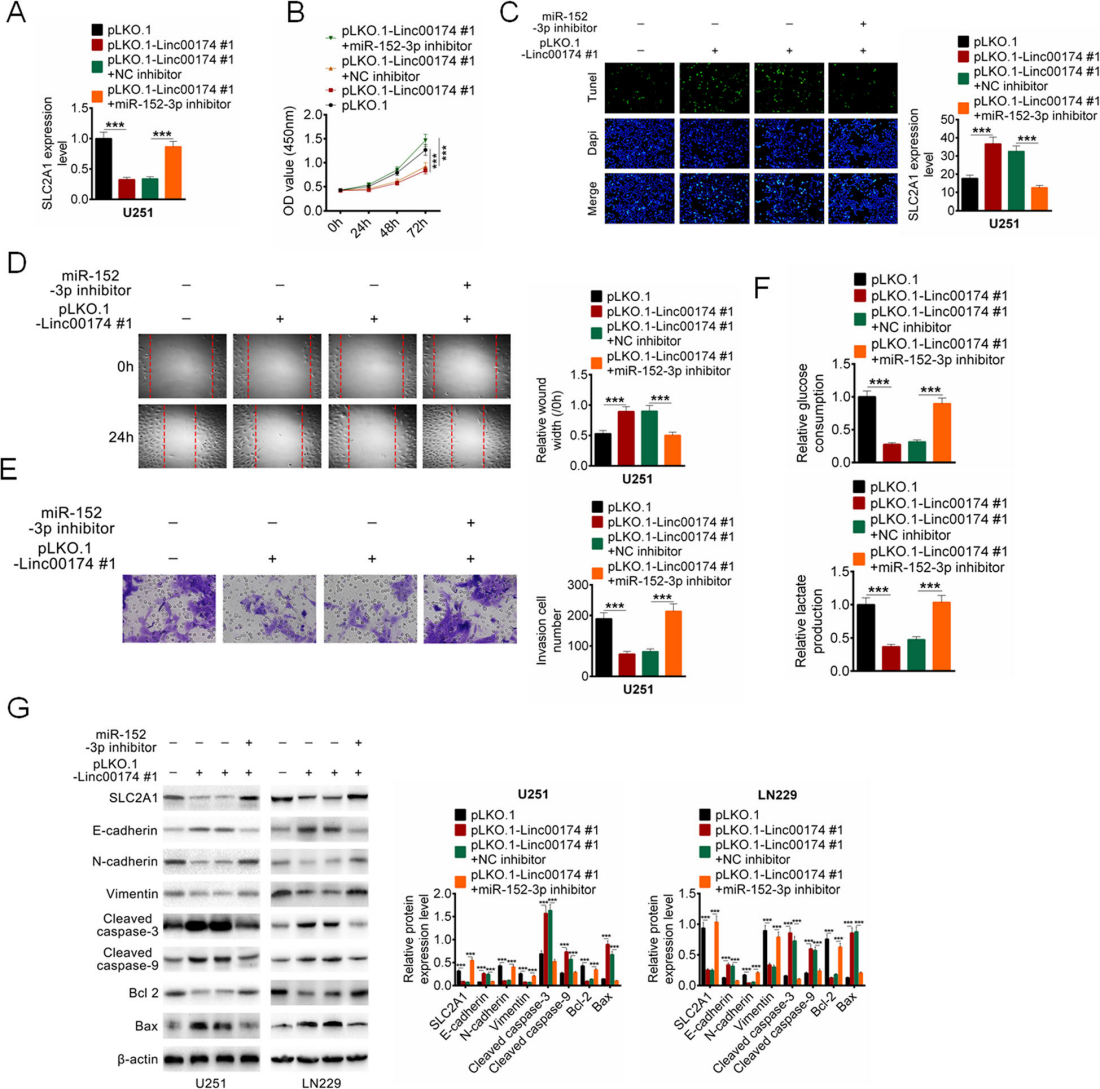
LINC00174 regulated cell phenotype of glioma cells by targeting miR-152-3p. **a** U251 cells were transfected with pLKO.1, or pLKO.1-LINC00174#1, or pLKO.1-LINC00174#1 + miR-NC, or pLKO.1-LINC00174#1 + miR-152-3p inhibitor, the expression of SLC2A1 was detected by RT-qPCR. **b**-**f** Cell proliferation, apoptosis, migration, invasion and glycolysis were then identified by CCK8, TUNEL, wound healing, transwell and ELISA, respectively. **g** Protein expression of SLC2A1, E-cadherin, N-cadherin, Vimentin, Cleaved caspase-3, Cleaved caspase-9, Bcl-2, and Bax were examined by western blot analysis. Data are presented as the mean ± SD. ***P < 0.001

To further identify the underlying mechanism by which LINC00174 regulated cell activities in glioma cells, rescue assays were performed to further confirm that LINC00174 contributed to glioma progression by regulating miR-152-3p/SLC2A1 signal pathway. U251 cells were transfected with pLKO.1, or pLKO.1-LINC00174#1, or pLKO.1-LINC00174#1 + miR-NC, or pLKO.1-LINC00174#1 + miR-152-3p inhibitor. Cell proliferation, apoptosis, migration, invasion and glycolysis were then identified by CCK8, TUNEL, wound healing, transwell and ELISA, respectively. It was found that SLC2A1 expression was increased in U251 cells with pLKO.1-LINC00174#1 + miR-152-3p inhibitor transfection (*P* < 0.001, Fig. 6a). as shown in Fig. 6b-f, LINC00174 knockdown effectively inhibited cell proliferation, migration, invasion and glycolysis, and increased cell apoptosis of U251 cells compared with the cells with pLKO.1 tranfection, and miR-152-3p inhibitor transfection significantly reversed the inhibiting effect of LINC00174 knockdown on U251 cells (*P* < 0.001, Fig. 6b-f). Besides, the expressions of SLC2A1, E-cadherin, N-cadherin, Vimentin, Cleaved caspase-3, Cleaved caspase-9, Bcl-2, and Bax were identified by western blot analysis. LINC00174 knockdown evidently regulated the protein expression, while miR-152-3p inhibitor effectively abolished the effect of LINC00174 knockdown on protein expression (Fig. 6g).

## Discussion

Gliomas are the most common malignant tumors in the central nervous system, and the exact pathogenesis of which is still unclear. LncRNA is a kind of non-protein-coding RNA, which plays an indispensable role in the occurrence and development of various tumors. Shen et al. reported that lncRNA LINC00174 was overexpressed in colorectal cancer samples and cells, and abnormal expression of LINC00174 indicated a poor prognosis of colorectal cancer patients. In the present study, we first identified the expression of LINC00174 in the glioma tissues and cells. We found that LINC00174 was overexpressed in the glioma tissues and cells, and high expression of LINC00174 showed a unfavourable prognosis in glioma patients. The effect of LINC00174 on cell proliferation, apoptosis, migration and invasion was also examined, and the results exhibited that LINC00174 knockdown effectively inhibited cell proliferation, migration and invasion, and promoted cell apoptosis of U251 and LN229 cells. Furthermore, LINC00174 overexpression accelerated cell proliferation, migration and invasion, and decreased cell apoptosis of U251 and LN229 cells. In addition, the silencing LINC00174 could delay tumor growth in vivo. These data reveals that LINC00174 acts as an oncogene in glioma and facilitates the progression of glioma.

We subsequently explored the targeted miRNA of LINC00174 by biological analysis, and miR-152-3p was predicted to combine with LINC00174, which was also verified by RIP, RNA pull down and dual luciferase reporter analysis. A number of studies reported that miR-152-3p expression was decreased in glioma [22], breast cancer [23], hepatic carcinoma [24], malignant melanoma [25], and prostate cancer [26]. Overexpression of miR-152-3p showed anti-tumor effect on cancer cells [26]. Sun et al. reported that miR-152-3p was down-regulated in glioma samples and inhibited cell proliferation and invasion by suppressing the expression of DNMT1 [22]. In the present study, we found that miR-152-3p could interact with LINC00174, and miR-152-3p expression was negatively correlated with LINC00174 expression in glioma clinical samples. Moreover, siLINC00174 attenuated cellular activities of glioma cells, while miR-152-3p inhibitors evidently reversed the anti-tumor effect of siLINC00174 on glioma cells. The above results investigate that LINC00174 regulates cell phenotype of glioma cells via targeting miR-152-3p.

The target mRNA of miR-152-3p was afterwards examined. SLC2A1 was the downstream target of miR-152-3p. SLC2A1 is a ratelimiting transporter for glucose uptake, and plays a crucial role in glycolysis. Cancer cells characterized by rapid proliferation require more energy produced by glycolysis than normal cells. Previous studies reported that SLC2A1 expression was up-regulated in non-small cell lung cancer [27], colon cancer [28], and gastric cancer [20], and mediated the glucose transport in cancer cells. Chen et al. reported that cAMP responsive element binding protein 1 affected glucose transport in glioma cells by regulating the expression of GLUT1 (SLC2A1), and mediated the metabolism and progression of glioma [29]. In this work, it was found that SLC2A1 was overexpressed in glioma samples, and SLC2A1 expression was negatively associated with miR-152-3p expression in glioma patients. By a series of cellular functional experiments, we demonstrated that LINC00174 could promote the glycolysis in glioma cells. To further identify whether LINC00174 facilitates the glycolysis by regulating miR-152-3p/SLC2A1 pathway, the rescue experiments were performed. The results displayed that miR-152-3p mimic inhibited cell proliferation, migration, invasion and glycolysis in glioma cells, while SLC2A1 knockdown abolished the effect of miR-152-3p mimic on glioma cells. The results reveal that LINC00174 promotes glioma cell proliferation, migration, invasion and glycolysis through regulating miR-152-3p/SLC2A1 axes. Furthermore, the expressions of SLC2A1, E-cadherin, N-cadherin, Vimentin, Cleaved caspase-3, Cleaved caspase-9, Bcl-2, and Bax were identified by western blot analysis. E-cadherin, N-cadherin, and Vimentin are important factors participating in the epithelial-mesenchymal transition (EMT), which promotes the migration and invasion of cells [30]. As well known, Cleaved caspase-3, Cleaved caspase-9, Bcl-2, and Bax play crucial role in cell apoptosis [31]. LINC00174 knockdown evidently regulated the protein expression, while miR-152-3p inhibitor effectively abolished the effect of LINC00174 knockdown on protein expression. The results indicate that LINC00174 adjust cellular activities by regulating these proteins.

The highlights of our study are: (1) LINC00174 was overexpressed in glioma. (2) LINC00174 predicted an unfavorable prognosis in glioma patients. (3) LINC00174 promoted glycolysis and tumor progression by targeting miR-152-3p/SLC2A1 axes. Although a lot of studies demonstrates that SLC2A1 mediated the glucose transport in cancer cells, few studies focus on the detail function and the mechanism of SLC2A1 in glioma, and the pathways related with SLC2A1 in cancer progression are also rarely studied. Systematic study concerning the biological function and mechanism of SLC2A1 in glioma will be an important part of our future studies.

## Conclusions

In general, our study investigated that LINC00174 expression was increased in glioma tissues and cell lines. High expression of LINC00174 predicted an unfavorable prognosis in glioma patients. LINC00174 promoted cell proliferation, migration, invasion and glycolysis via sponging miR-152-3p and up-regulating SLC2A1 expression. These results and findings provide a novel insight for glioma diagnosis and treatment.

## Data Availability

The data used in this study is available on a reasonable request from the corresponding author.

## Abbreviations

CCK-8: Cell Counting Kit-8
CRC: Colorectal cancer
EMT: Epithelial-mesenchymal transition
FBS: Fetal bovine serum
GLUT1: Glucose transporter 1
lncRNA: Long-chain non-coding RNA
NHAs: Normal human astrocytes
PTBE: Peritumoral brain edema
RT-qPCR: Reverse transcription-quantitative polymerase chain reaction
shRNA: Short hairpin RNA
siRNA: Small interfering RNAs
SLC2A1: Solution vector family 2 promotes glucose transporter 1
TCGA: The Cancer Genome Atlas
